# Highly selective generation of vanillin by anodic degradation of lignin: a combined approach of electrochemistry and product isolation by adsorption

**DOI:** 10.3762/bjoc.11.53

**Published:** 2015-04-13

**Authors:** Dominik Schmitt, Carolin Regenbrecht, Marius Hartmer, Florian Stecker, Siegfried R Waldvogel

**Affiliations:** 1 Institute for Organic Chemistry, Johannes Gutenberg University Mainz, Duesbergweg 10–14, 55128 Mainz, Germany; 2 BASF SE, GCN/ES—M311, 67056 Ludwigshafen, Germany

**Keywords:** adsorption, electrochemistry, lignin, nickel, renewable resources

## Abstract

The oxidative degradation of lignin into a variety of valuable products has been under investigation since the first half of the last century. Especially, the chance to claim this cheap, abundant and renewable source for the production of the important aroma chemical vanillin (**1**) was one of the major driving forces of lignin research. So far most of the developed methods fail in technical application since no viable concept for work-up is included. This work represents a combined approach of electrochemical conversion of Kraft lignin and product recovery by adsorption on a strongly basic anion exchange resin. Electrolysis conditions are optimized regarding reaction temperatures below 100 °C allowing operation of aqueous electrolytes in simple experimental set-up. Employing ion exchange resins gives rise to a selective removal of low molecular weight phenols from the strongly alkaline electrolyte without acidification and precipitation of remaining lignin. The latter represents a significant advantage compared with conventional work-up protocols of lignin solutions.

## Introduction

**Scheme 1:**
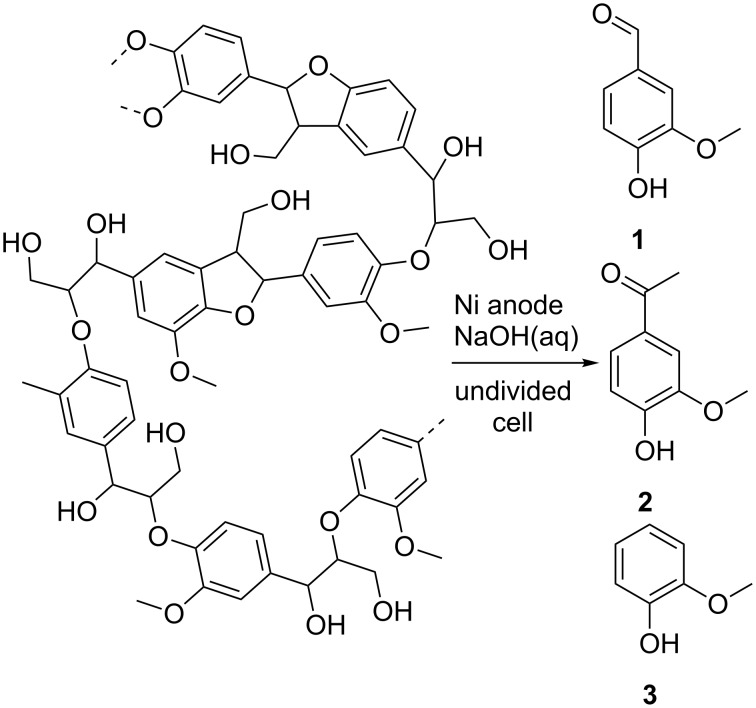
Direct electrochemical degradation of lignin into low molecular weight phenolic compounds.

The biopolymer lignin is one of the most abundant and renewable feedstocks in the world [1–3]. Moreover, lignin represents the largest source of aromatic compounds among renewables and can be considered as non-food biomass. It usually occurs as a major waste fraction of the pulping industry on a multimillion ton scale [4]. This source has the potential to be an alternative for petroleum-based production of fuels as well as fine chemicals [5–7]. Since the middle of the last century, the large amount of aromatic structural features making up the polymer led to much effort concerning efficient degradation methods into high value fine chemicals like vanillin (**1**), acetovanillone (**2**) and guaiacol (**3**) (Scheme 1) [8–10]. Different approaches for selective degradation of lignin, applying catalytic, microbacterial, photochemical, sono-chemical and electrochemical methods were investigated but struggled with several problems [11–17]. The dominating challenges are usually low selectivity resulting in a plethora of products, drastic and technically unreasonable reaction conditions, purification of the resulting crude product mixture, and separation of the desired products from unreacted lignin [18–20]. When using transition metal catalysts they commonly disappear in the unreacted lignin contaminating that particular material which limits further subsequent use. Electrochemistry is one of the most promising approaches for highly sustainable conversions because only electrons serve as reagent [21–27]. Consequently, such conversions are considered as reagent-free and avoid reagent waste [28–30]. We present a highly selective electrochemical approach providing an almost exclusive formation of vanillin (**1**) under very mild reaction conditions. The application of a strongly basic anion exchange resin allows an elegant separation of the formed vanillin (**1**) from remaining lignin directly out of the basic reaction solution.

## Results and Discussion

The electrochemical degradation of lignin in alkaline media is usually performed on nickel anodes. Utley et al. presented a very promising electrochemical approach using Ni anodes which enabled conversion of lignosulfonate in a filter press cell at elevated temperature and pressure. The conversion led to high yields of vanillin (**1**) in the range of 5–7 wt %. The complex experimental set-up as well as evolution of hydrogen are a major drawback of this approach [31]. Nevertheless, the applied Ni electrodes usually exhibit a high stability against corrosion at these conditions due to the formation of an electrocatalytic surface layer which is stable in alkaline electrolytes [32]. Kraft pulping represents the predominant pulping process [33]. Due to this we investigated the electrochemical degradation of Kraft lignin and avoided the use of lignosulfonate which originates from the outbounding sulfite process. The most common mechanistic rationale indicates the formation of an electrocatalytically active NiOOH species at the anodic surface which is regenerated during lignin oxidation [34]. The chemical relation between Ni and Co implies a similar electrocatalytic behaviour. The major focus of this study was to investigate the applicability of a lignin degradation process under technically relevant conditions. This implies an aqueous system due to the limited solubility of Kraft lignin as well as temperatures below 100 °C to avoid pressurized systems. Several electrode materials, based on Ni or Co alloys, were investigated towards their electrocatalytic activity in this particular degradation process. Table 1 displays yields of **1** by electrochemical degradation using the most productive anode materials.

**Table 1:** Influence of the anode material on the electrochemical degradation of lignin.^a^

Entry	Anode^b^	UNS-#	Alloy base	Yield of vanillin (**1**) / wt %^c^

1	Ni	–	–	0.7
2	Monel 400k	N04400	Ni	0.7
3	Nichem 1151	–	Ni	1.0
4	Co	–	–	1.4
5	Stellite 21	W73021	Co	1.8

^a^Electrolysis conditions: 80 °C, constant current (1.9 mA∙cm^−2^), undivided cell, 2688 C∙g^−1^, 0.525 g Kraft lignin. ^b^Detailed information about the alloys can be found in Supporting Information File 1. ^c^Based on used Kraft lignin. UNS = Unified numbering system.

**Figure 1:**
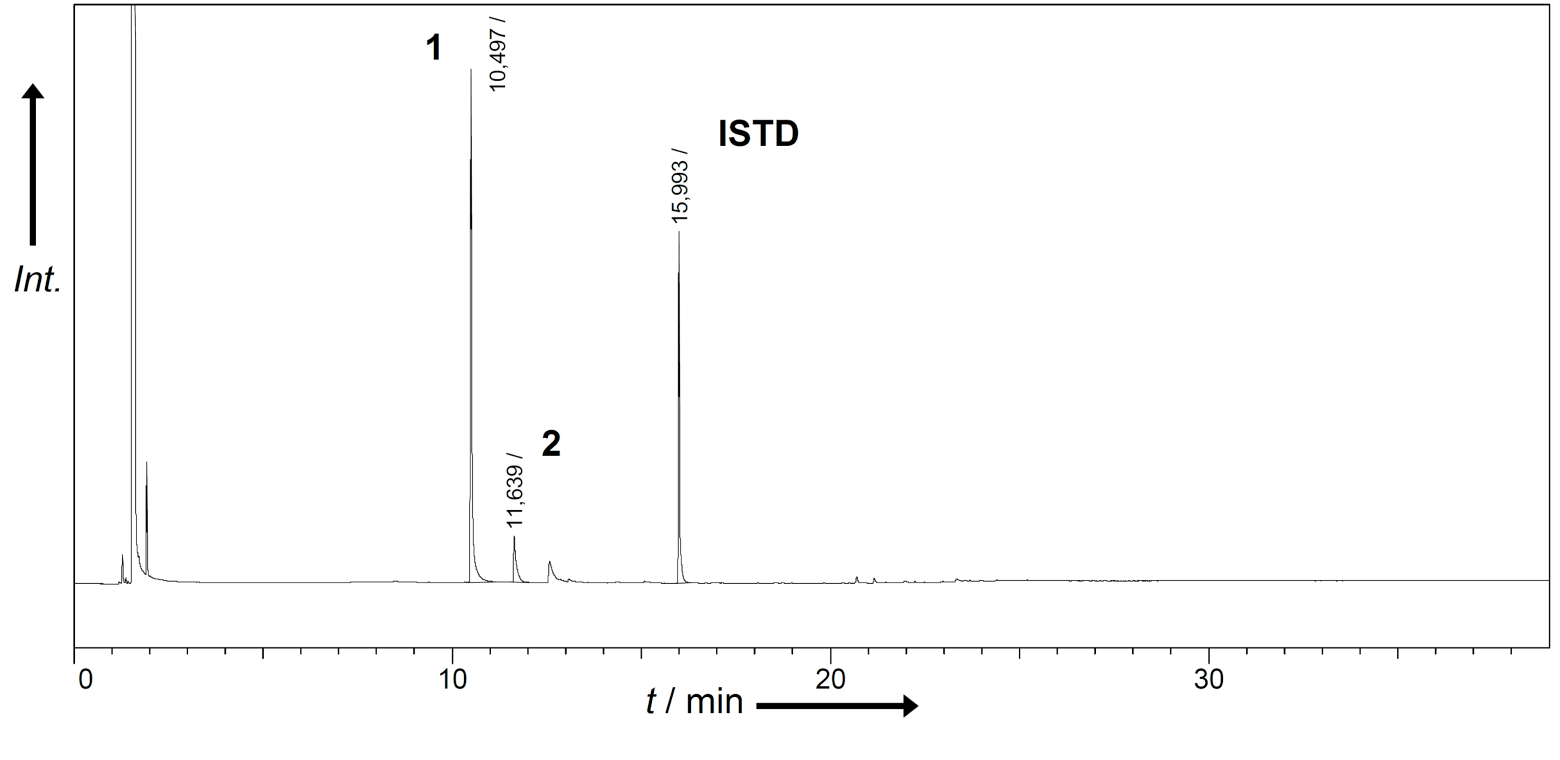
Crude product composition after electrochemical treatment of lignin at Ni-based electrodes by gaschromatography. Retention times: 10.50 min vanillin (**1**), 11.64 min acetovanillone (**2**), 16.00 min dodecylbenzene (ISTD).

Under the conditions described, the electrochemical process usually resulted in moderate yields of **1** <2.0 wt % per electrolysis run but the selectivity towards vanillin (**1**) formation is outstanding (Figure 1). The only other volatile byproduct formed in much lower yields compared to **1** is acetovanillone (**2**). Information about the quantities of **2** are given in Supporting Information File 2. In general, the application of Co-based materials resulted in higher yields of vanillin (**1**) with a maximum of 1.8 wt %. Unfortunately, all investigated Co-based alloys show some corrosion leading to mass loss and the concomitant formation of Co oxides found as dark coating on the electrode surface as well as suspended to a small extent in the electrolyte.

**Figure 2:**
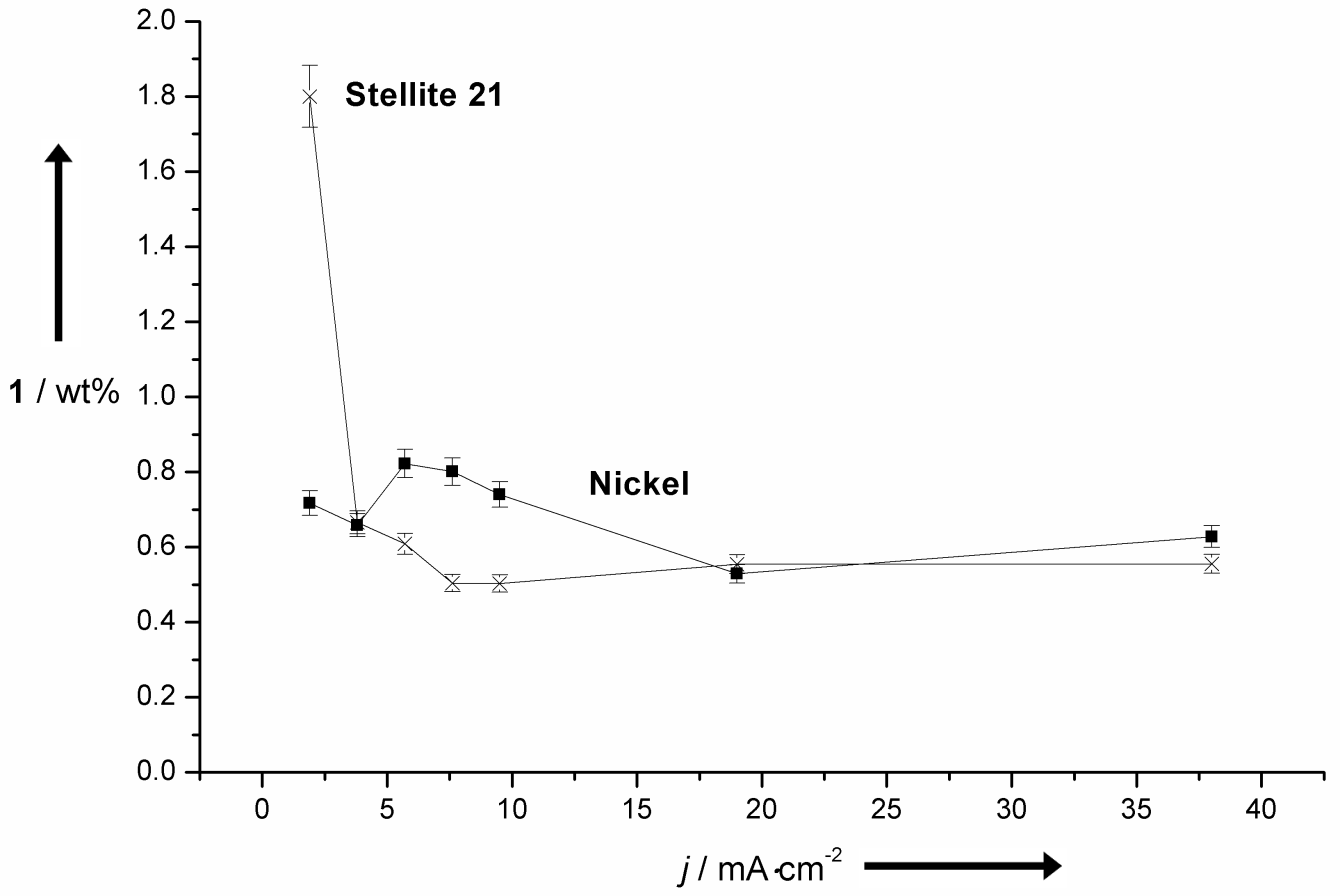
Influence of the current density onto the yield of **1** using Ni or Stellite 21 anodes.

**Figure 3:**
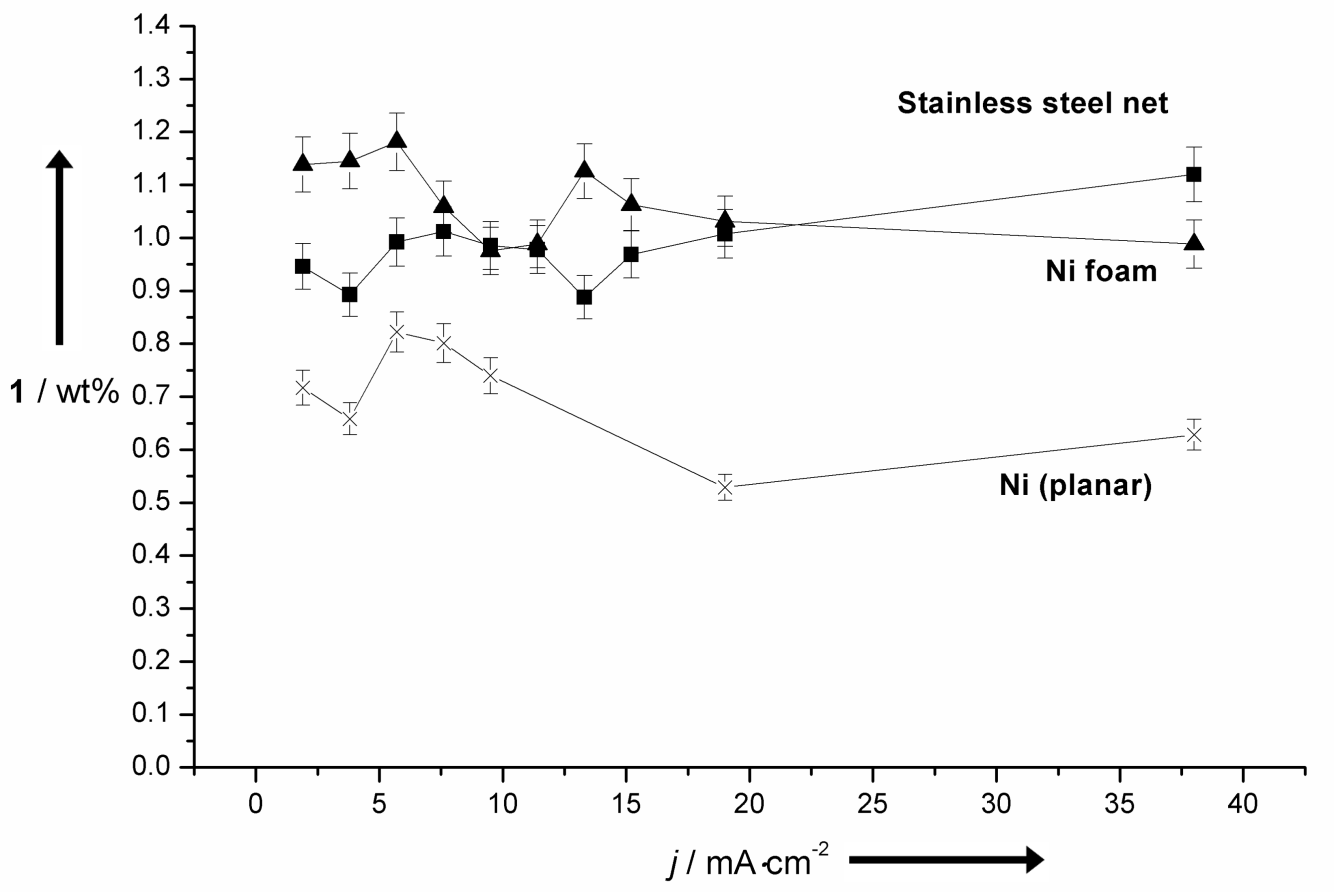
Influence of the current density on the yield of **1** using different geometries of anodic materials.

The application of Ni-based materials on the other hand results in lower yields up to 1.0 wt %, but no corrosion is observed. Besides the applied anode materials, the current density has a tremendous influence on the achievable yield. Rather low current densities <2.0 mA∙cm^−2^ usually result in the highest yields of vanillin (**1**) independent of the electrode materials. Especially, Co-based materials are very sensitive to this parameter and even a slight increase of the current density leads to a drastic drop in the yield of vanillin (**1**). This effect is displayed in Figure 2 comparing planar electrodes of Ni and the Co base alloy Stellite 21. Low current densities are very unfavourable from a technical point of view due to long electrolysis times. Three-dimensional electrodes are a suitable way to increase the effective anodic area leading to an improved space–time yield. For this reason 3D materials composed of different Ni-based materials were employed as electrode materials for the electrochemical process (Figure 3).

**Figure 4:**
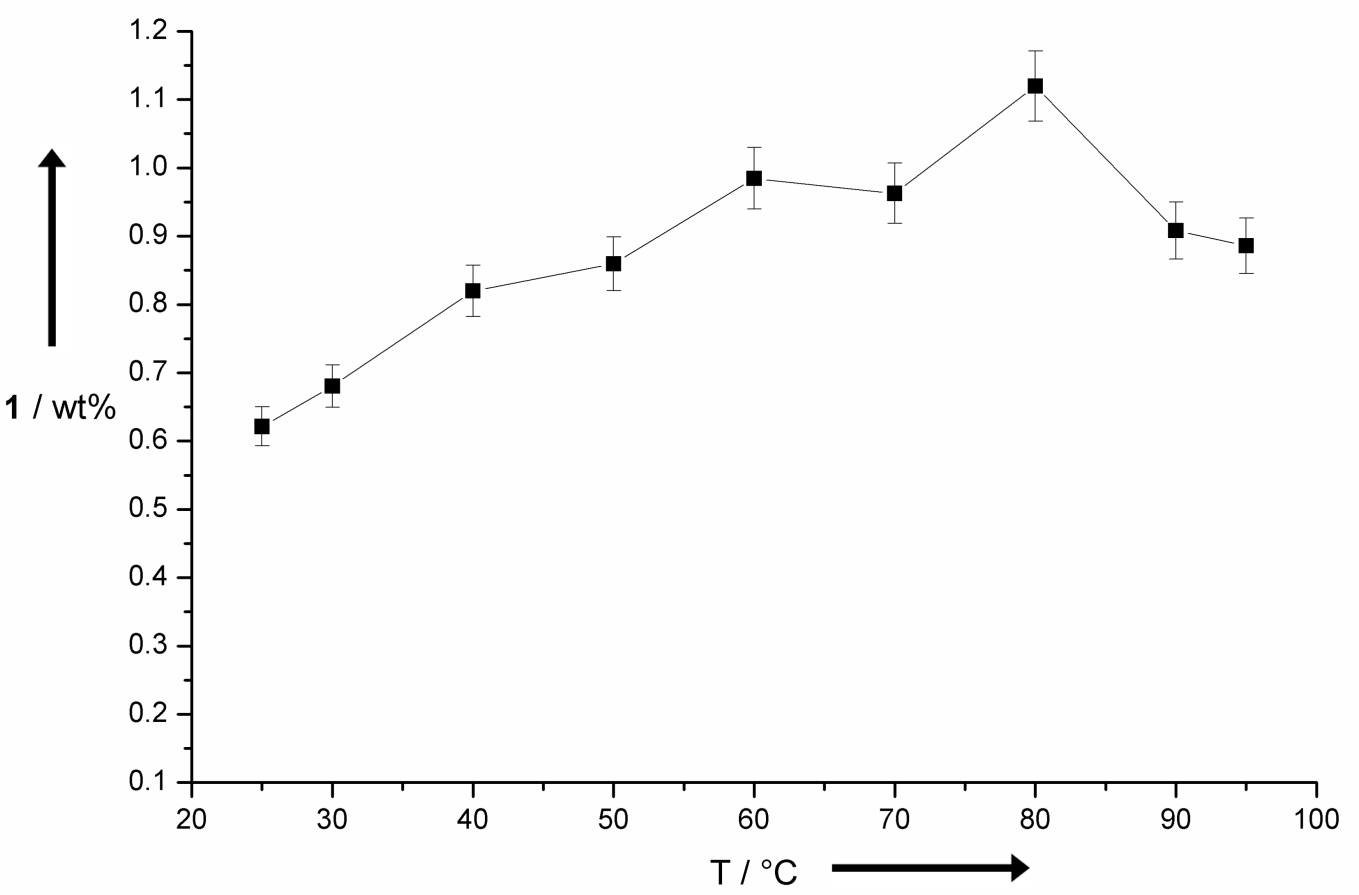
Influence of the reaction temperature onto anodic degradation of lignin using stainless steel electrodes.

**Figure 5:**
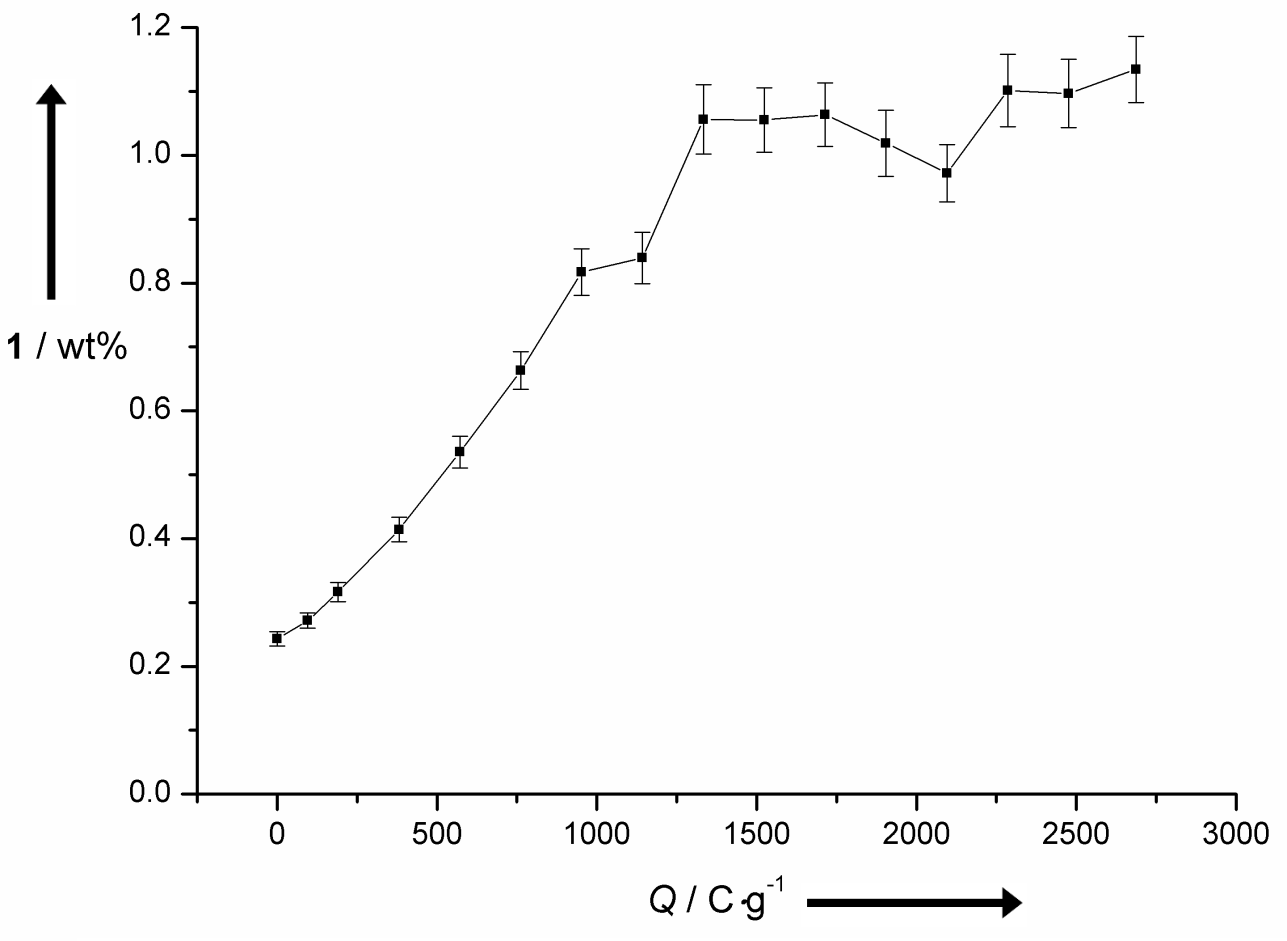
Influence of the applied current onto the yield of **1** by electrochemical degradation of lignin using stainless steel electrodes, applying a current density of 38 mA∙cm^−2^.

A comparison of 3D and plane Ni-based materials shows that even at low current densities of <2.0 mA∙cm^−2^ surface enhanced materials are superior to planar electrodes. Ni foam and stainless steel electrodes, with a Ni content of up to 13%, showed a very similar and promising behaviour especially at elevated current densities. With current densities of up to 38 mA∙cm^−2^ (this number corresponds to the geometric surface directly exposed to counter electrode) almost constant yields ≥1.0 wt % of **1** were observed. This application gives rise to increased yields of **1** as well as a decrease of electrolysis time to 5% of the initially applied with planar electrodes. Another important factor for the efficiency of the selective degradation to **1** is the reaction temperature. At elevated temperatures usually decoiling or fragmentation takes place in the lignin particles [35–36]. This is very important to improve the accessibility of possible reaction sites located rather inside the lignin particle to the anode surface and high temperatures >100 °C are usually chosen for efficient degradation processes. Due to limited solubility of the commonly used Kraft lignin in aqueous systems, it was necessary to set-up massive autoclaves [37]. This was always a very limiting aspect for technical realizations due to cost and safety issues. It is noteworthy, that on the cathode hydrogen is formed and performing the electrolysis in a closed system is not desired. However, even at rather low reaction temperatures between 20–80 °C the particle behaviour and also the yield of **1** is influenced tremendously (Figure 4). In the past, the use of different mediators and catalysts often led to the formation of over oxidation products, i.e., vanillic acid (**4**) [34]. Our system avoids the formation of these low value products even if an excess of current is applied. As depicted in Figure 5 an almost linear increase of **1** is observed until an applied current of about 1200 C∙g^−1^.

Further current does not lead to an increased formation of **1**. But the system tolerates the excess and the formed **1** is not consumed to generate oxidation products like vanillic acid (**4**). Under these conditions the electrochemical oxidation of vanillin (**1**) does not take place. This was proven by a control experiment trying to oxidize vanillin (**1**) in alkaline solution at Ni foam and stainless steel electrodes. In both cases no formation of vanillic acid **4** was observed and the starting material was recovered almost quantitatively which indicates that no oligomer formation took place (see Supporting Information File 2). Screening of different anode materials and reaction parameters allowed an optimization of the electrochemical process. Ni foam electrodes enable enhanced current densities of up to 38 mA·cm^−2^ without negative influence on the yield of **1**. A reaction temperature of 80 °C and an applied current of 1500 C·g^−1^ leads to the maximum yield of **1**.

With the ability to selectively generate vanillin (**1**) from lignin in hand, we turned our attention to the development of an isolation strategy for the product. As in the method discussed above, degrees of conversion were usually rather low, but this is compensated by the enormous scope of the feedstock lignin [35]. But selectivity and product recovery are the most challenging aspects. After the electrolysis the electrolyte contains large amounts of unreacted, respectively chemically modified lignin. A conventional approach for product recovery includes acidification of the mixture, which leads to precipitation of lignin. Filtration followed by liquid–liquid extraction results in the clean product **1**. This procedure is rather problematic from a technical point of view. Filtration processes usually are time and maintenance intensive processes but even more disadvantageous is acidification of the whole electrolyte. That approach is expensive comparing the amount of acid necessary to neutralize the solution and the moderate yields of **1** which can be achieved by these processes. Consequently, alternative concepts are necessary to allow product removal without precipitation of lignin and acidification of the whole reaction mixture.

**Figure 6:**
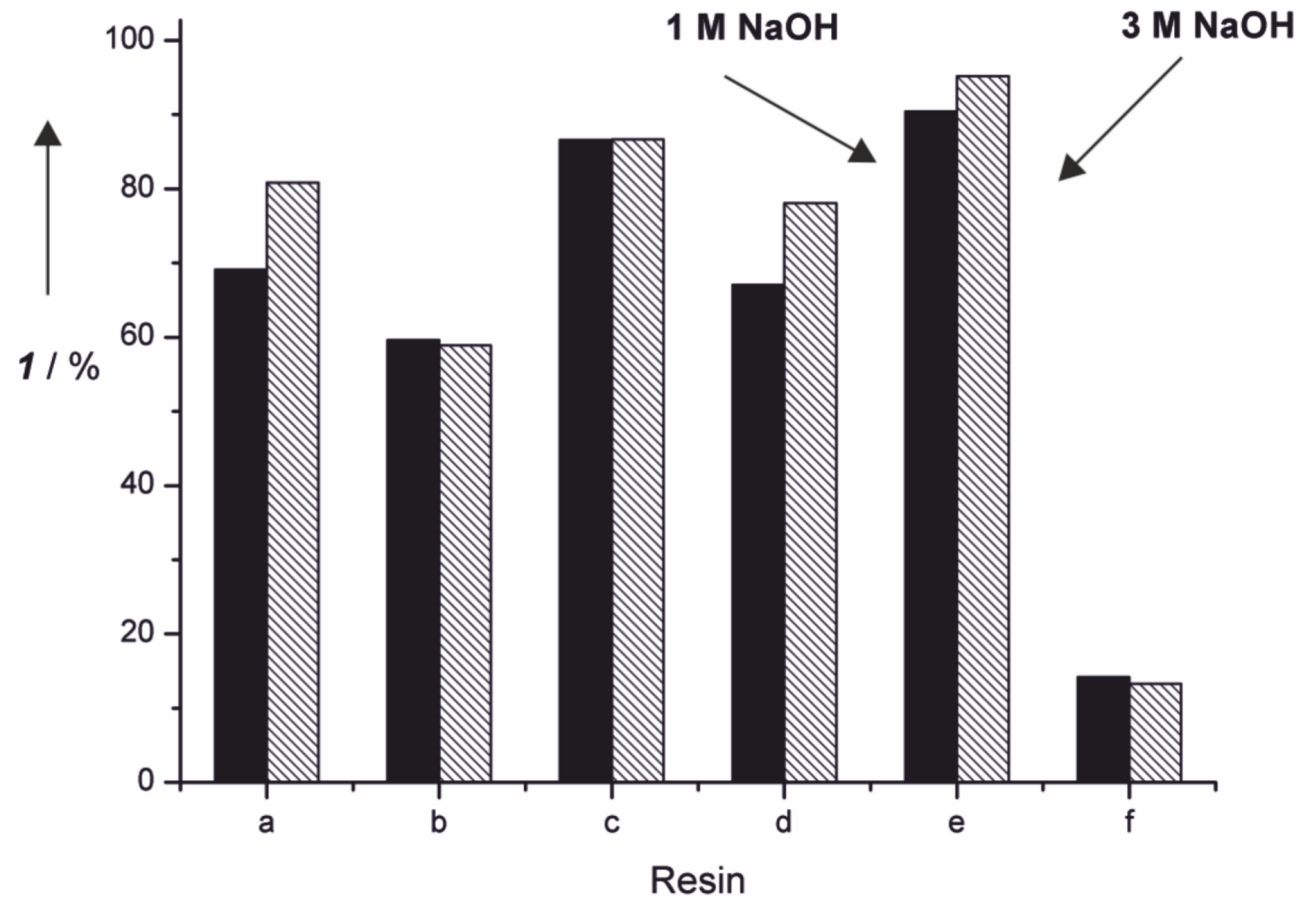
Amount of vanillin (**1**) removed by adsorption in a batch process at different strongly basic anion exchange resins. Experiments were performed at two different NaOH concentrations and desorption was realized by acidic treatment of the loaded resins.

For this purpose the applicability of strongly basic anion exchange resins was tested. It is known from literature that these resins can be utilized for phenol recovery from waste water streams at different pH [38]. These methods usually take advantage of the combined physi- and ionosorptive interactions between the resin and the adsorptive phase. In the case of phenolate stronger, ionic interactions usually dominate at basic pH [39]. But even under acidic conditions strong interactions between the polymer backbone and the adsorptive phase remain [40]. For this reason several commercially available resins were tested concerning their adsorption and desorption affinity towards **1** in model solutions at two different pH values (Figure 6). The different resins with their functionalities and the individual polymeric backbone are listed in Table 2.

**Table 2:** Polymeric backbones and functionalities of the different strongly basic anion exchange resins used for batch adsorption experiments.

Resin^a^	Backbone	Ionic function

**a**	Polyvinylpyridine divinylbenzene	*N*-Methylpyridinium
**b**	Polystyrene divinylbenzene	Tetraalkylammonium
**c**	Polystyrene divinylbenzene	Tetraalkylammonium
**d**	Polystyrene divinylbenzene	Tetraalkylammonium
**e**	Polystyrene divinylbenzene	Tetraalkylammonium
**f**	Polyacrylate divinylbenzene	Tetraalkylammonium

^a^Commercial names of the different resins, corresponding exchange capacities and further information about specifications of the resins are listed in Supporting Information File 1.

**Figure 7:**
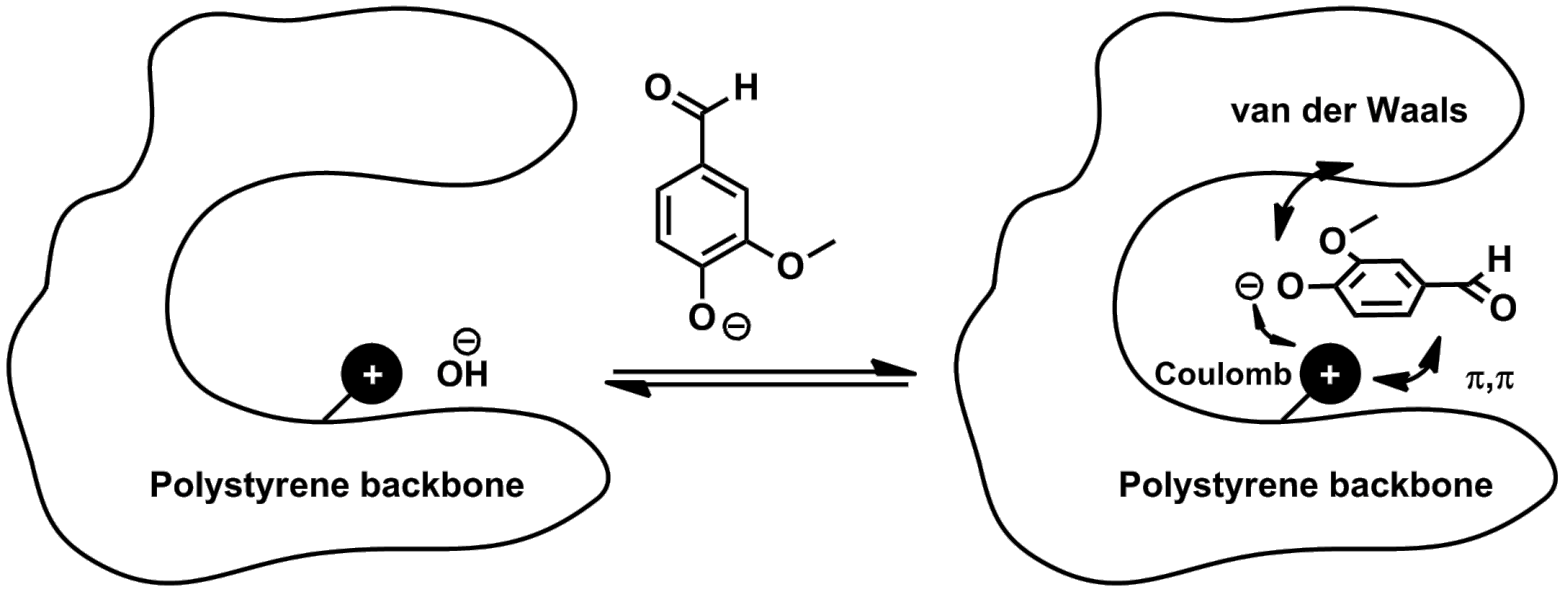
Different attractive interactions between ion exchange resin and the vanillate anion.

Even in batch processes it was possible to remove more than 90% of dissolved **1** from the model solution which indicates that strong interactions between the resins and the adsorptive phase takes place. Desorption of the product can easily be performed by acidic treatment of the loaded resins. The most promising desorption system so far is a solution of EtOAc and AcOH (ratio 8:2). This treatment leads to protonation of vanillate anions adsorbed at the resin and ionic interactions between the resin and the product vanish. The remaining interactions between vanillin (**1**) and the aromatic backbone are not strong enough to prevent dissolution of **1** in the eluent. The results indicate that besides the ionic function the polymeric backbone has a very important influence on the adsorption behavior (Figure 7).

**Figure 8:**
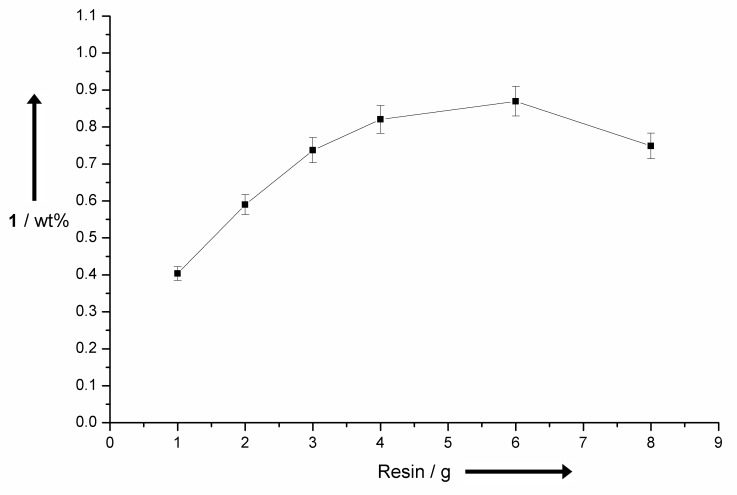
Recovery of vanillin (**1**) by adsorption from lignin containing reaction solutions after electrochemical treatment at Ni foam electrodes. Different amounts of resin were applied in a batch process. Desorption was performed treating the loaded resins with an acidic solution (EtOAc/AcOH, 8:2) in a batch process.

The polystyrene backbones appear to be especially well suited for the adsorption of **1** from alkaline solutions. This can be explained by attractive π–π interactions between the backbone and the adsorptive phase. All resins containing an aromatic backbone lead to a loading of **1** >50% based on the total amount of used **1**. Resin **f** is a polyacrylate resin. This resin showed a far inferior loading of **1** <20% which supports the assumption that the polymeric backbone has a major importance for the adsorption process. Control experiments of non-modified polystyrene resin gave no adsorption at all. These batch experiments were optimized regarding the low vanillin (**1**) concentrations in the corresponding reaction solution after electrochemical degradation of lignin. Therefore, experiments were performed applying a high ratio of resin to vanillin (**1**). Further studies regarding the total capacity of this resin were performed showing that a loading of more than 60% is possible. This allows an easy removal of vanillin (**1**) on a gram scale (see Supporting Information File 2). The superior resin **e** was used for the adsorption of **1** from the lignin-containing reaction mixture. Electrochemical degradation reactions of lignin were performed and afterwards various amounts of anion exchange resin were added to perform a batch process for adsorption of **1** (Figure 8).

**Figure 9:**
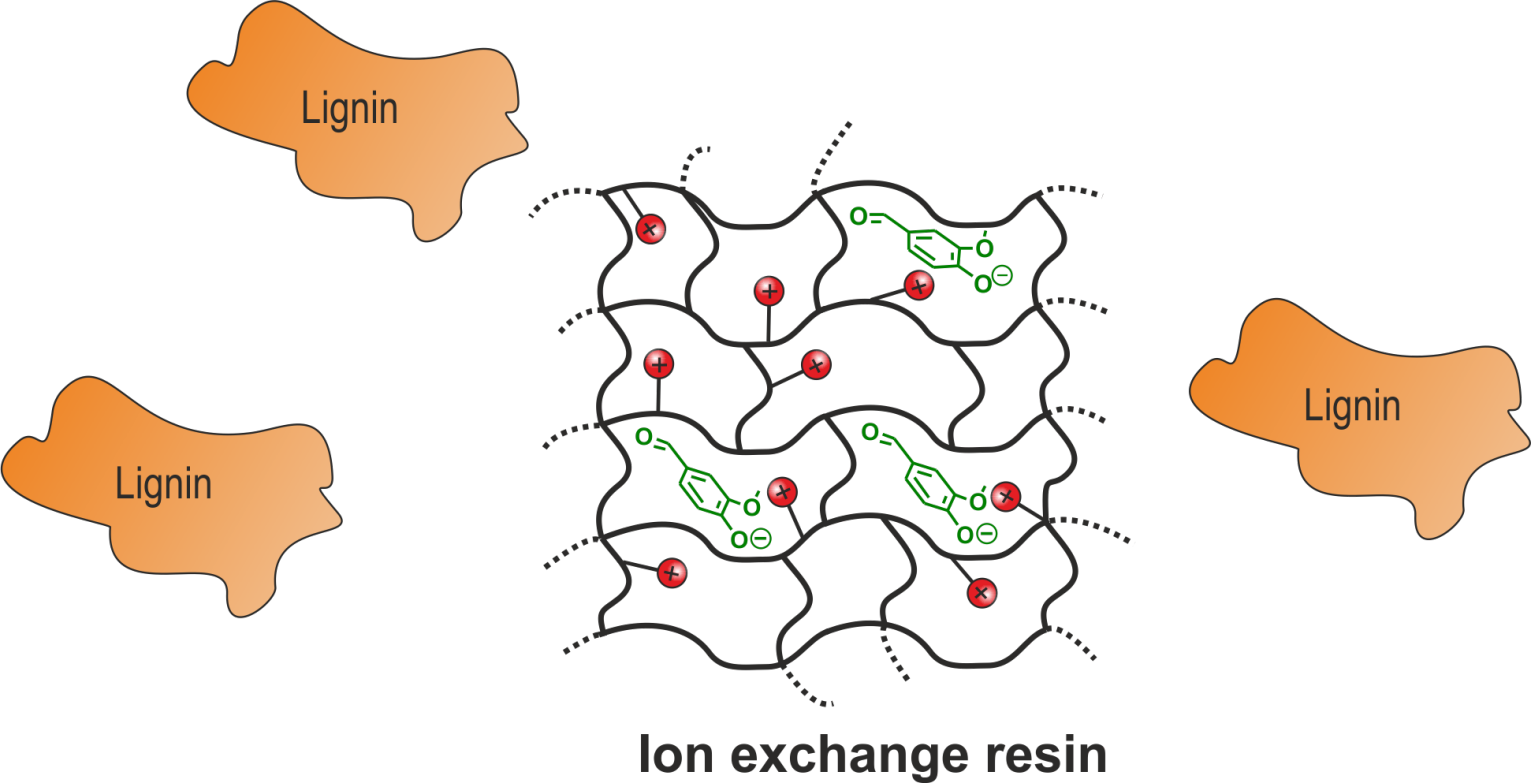
Adsorption of vanillin (**1**) on anion exchange resins and size exclusion of lignin particles by application of gel type resins.

The results indicate that a large excess of resin is necessary to adsorb the maximum amount of **1**. Applying 6 g of resin led to the maximum yield of vanillin (**1**) of 0.9 wt % using this work-up protocol. This is close to the theoretical maximum yield of 1.0 wt % which was observed by conventional work-up of the reaction solution. Addition of more than 6 g of resin does not lead to an enhanced product removal, even lower yields of **1** were observed. This behavior can be rationalized by residual loading of the resin. The concentration of **1** in the reaction solution is about 0.06 mg·mL^−1^ and even under acidic conditions interactions between adsorptive phase and backbone are strong enough to keep a certain amount of formed **1** adsorbed in the equilibrium. This incomplete desorption is a common problem of batch processing [41]. To avoid this process it is necessary to allow a continuous shift of the equilibrium which can be realized by a continuous adsorption and desorption process. This was realized by setting up a column filled with anion exchange resin and the corresponding solutions for adsorption (lignin containing reaction mixture) and desorption (acidic eluent) of **1** were pumped through the column. This set-up allows a continuous enrichment of **1** on the column which avoids acidification of the solution and no precipitation occurs. The performance and applicability of this process was investigated by ten identical electrochemical degradation reactions followed by adsorption of the resulting solutions in a continuous process on the same column. After adsorption the depleted reaction solutions were analyzed for residual amounts of **1** by conventional work-up. No fraction of the depleted solution showed any content of vanillin (**1**) which indicates a complete take up of **1**. Afterwards the loaded resin was treated using an acidic eluent consisting of EtOAc/AcOH (8:2) analogous to the continuous adsorption process. Afterwards the acidic fraction was investigated concerning its content of **1**. It was observed that the expected maximum yield of 1.0 wt %, based on the total amount of used Kraft lignin, was exceeded and an effective yield of 1.3 wt % was found. This surprising observation can be explained by the optimized recovery process which avoids the very disadvantageous precipitation of lignin. The precipitate can include and adsorb certain amounts of **1** which leads to a reduced total yield. The present adsorption process avoids such precipitation and is by far superior to known conventional work-up procedures for alkaline lignin solutions. Using the desorption system EtOAc:AcOH (8:2) is very advantageous from an ecological point of view. Both components are environmental friendly and biocompatible. This is another advantage of this protocol compared with the conventional work-up procedure including acidification and liquid–liquid extraction applying dichloromethane as extracting agent. Excess of EtOAc and AcOH used for desorption can easily be recovered by distillation. Furthermore, the applied resin avoids adsorption of the dissolved lignin particles by size exclusion pictured in Figure 9. The chosen gel type resin is distinguished from macroreticular adsorbents by lower pore diameters [42–43]. Average pore diameters of gel type resins are in the range of 1–2 nm compared with macroreticular diameters up to several hundred nanometer. Lignin particles themselves can have larger diameters up to a few micrometers which allows an exclusion of these particles by application of gel type ion exchange resins [44]. So far no long-term study on the reusability of the resin was performed but the activity of the resin after an adsorption–desorption cycle with high loading of vanillin (**1**) was investigated and no loss of activity was observed. This indicates that adsorption of vanillin (**1**) has no negative influence on stability of the ion exchange resin (see Supporting Information File 2).

## Conclusion

In conclusion, our approach combines a highly selective electrochemical formation of vanillin (**1**) and a novel as well as viable work-up concept exploiting strongly basic anion exchange resins. As renewable feedstock we employed alkaline lignin solutions. Alloys of cobalt and nickel as anodic material are suitable forming in situ electrochemically active MO(OH) coatings. Despite higher yields for **1** using Co based anodes, Ni-based anodes seem to be the electrodes of choice due to their enhanced stability against corrosion. The electrolysis was successfully optimized to a process that can be operated below 100 °C and the application of 3D electrode designs, increased the space–time yield tremendously. To circumvent the common challenges in the work-up of lignin solutions (precipitation of lignin, use of excess acid and subsequent extraction of the low molecular weight products), we developed a novel and viable strategy for direct and selective removal of vanillin (**1**) and related phenols from the alkaline electrolyte. An easy to perform enrichment of the desired product by adsorption at strongly basic anion exchange resins was established. The larger lignin particles were not bound to the gel-type resins because the size exclusion effect does not affect them. Only small amounts of acid will be required to rinse the product from the solid support, which is of significant advantage to the conventional approaches. The resulting lignin-containing waste streams could be incinerated for energy production and base recovery performed, as currently done with waste streams of the pulping process [45]. The combined concepts represent a starting point for vanillin (**1**) production based on renewable resources [46–47].

## Supporting Information

File 1 Information about materials.

File 2 Experimental information.
