# Supplementary material for: Highly selective generation of vanillin by anodic degradation of lignin: a combined approach of electrochemistry and product isolation by adsorption
Source: Beilstein J Org Chem. 2015 Apr 13;11:473–80. doi: 10.3762/bjoc.11.53 (PMC4419547; doi:10.3762/bjoc.11.53)
Supplement: File 1 — Information about materials. [file Beilstein_J_Org_Chem-11-473-s001.pdf]

Supporting Information File 1

for

Highly selective generation of vanillin by anodic degradation of lignin: a combined approach of electrochemistry and product isolation by adsorption

Dominik Schmitt[1], Carolin Regenbrecht[1,2], Marius Hartmer[1], Florian Stecker[2], and Siegfried R. Waldvogel*[1]

Address: [1] Institute for Organic Chemistry, Johannes Gutenberg University Mainz, Duesbergweg 10 – 14, 55128 Mainz (Germany) and [2] BASF SE, GCN/ES—M311, 67056 Ludwigshafen (Germany)

Email: Siegfried R. Waldvogel - waldvogel@uni-mainz.de

* Corresponding author

Information about materials

Electrode materials

The applied Ni foam is displayed in Figure S1.

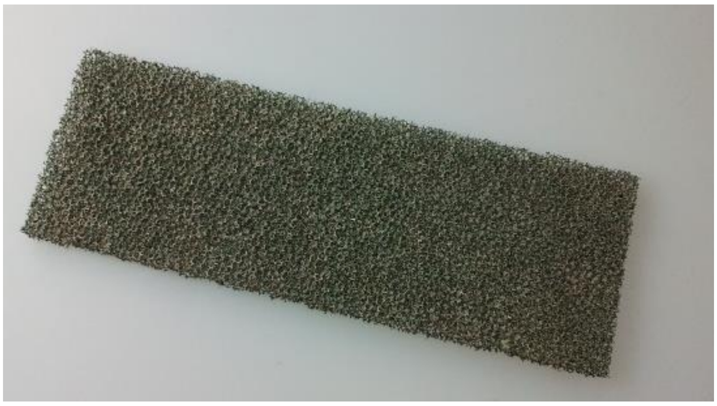

Figure S1: Ni foam electrode applied for the electrochemical degradation of lignin.

Characteristics of further electrode materials applied for the electrochemical degradation of lignin are listed in Table S1.

Table S1: **Applied electrode materials and compositions of corresponding alloys.**

Entry	Anode	UNS-#	Alloy base	Alloy Compostion / %
1	Ni (planar)	-	-	-
2	Monel 400k	N04400	Ni	C 0.3/Mn 2.0/Fe 2.5/Si 0.5/Cu 28.0-34.0
3	Nichem 1151 (planar)	-	Ni	P 11.6 – 11.8
4	Co (planar)	-	-	-
5	Stellite 21 (planar)	W73021	Co	Cr 26.0-29.0/Mo 4.5-6.0/C 0.2-0.4/Ni 2.0-3.0/Fe,Si,Mn traces
6	Ni foam	-	-	-
7	Stainless steel net (V4A)	S31603	Fe	C≤0.03/Si≤1.0/Mn≤2.0/Cr 16.5-18.5/Mo 2.0-2.5/Ni 10.0-14.0

Strongly basic anion exchange resins

Different strongly basic anion exchange resins were applied for the adsorption of vanillin in batch experiments as wells as in continuous approaches which are listed in Table S2.

Table S2: Strongly basic anion exchange resins for vanillin adsorption.

Resin	Commercial name	Ionic function	Particle size (diameter) [mm]	Exchange capacity [meq/mL]
a[A]	Reillex HPQ IE Resin	*N*-Methylpyridinium	0.3 - 1.0	>4.00
b[B]	Amberlite IRA 910 Cl	Tetraalkylammonium	0.5 - 0.8	1.00
c[B]	Amberlite IRA 402 OH	Tetraalkylammonium	0.7 – 0.9	0.95
d[B]	Ambersep 900 OH	Tetraalkylammonium	n.a.	0.80
e[B]	Dowex Monosphere 550a OH	Tetraalkylammonium	0.5 – 0.6	1.10
f[C]	Applexion XA 4141 Cl	Tetraalkylammonium	n.a.	0.80

[A] Polyvinylpyridine divinylbenzene backbone. [B] Polystyrene divinylbenzene backbone. [C] Polyacrylate divinylbenzene backbone. n.a. = Not available.
