# Supplementary material for: Highly selective generation of vanillin by anodic degradation of lignin: a combined approach of electrochemistry and product isolation by adsorption
Source: Beilstein J Org Chem. 2015 Apr 13;11:473–80. doi: 10.3762/bjoc.11.53 (PMC4419547; doi:10.3762/bjoc.11.53)
Supplement: File 2 — Experimental information. [file Beilstein_J_Org_Chem-11-473-s002.pdf]

Supporting Information File 2

for

Highly selective generation of vanillin by anodic degradation of lignin: a combined approach of electrochemistry and product isolation by adsorption

Dominik Schmitt[1], Carolin Regenbrecht[1,2], Marius Hartmer[1], Florian Stecker[2], and Siegfried R. Waldvogel*[1]

Address: [1] Institute for Organic Chemistry, Johannes Gutenberg University Mainz, Duesbergweg 10 –14, 55128 Mainz (Germany) and [2] BASF SE, GCN/ES—M311, 67056 Ludwigshafen (Germany)

Email: Siegfried R. Waldvogel - waldvogel@uni-mainz.de

* Corresponding author

Experimental information

General information

All reagents were used in analytical grades. For electrochemical reactions Ni foam, (AQUA TITAN, Dortmund, Germany) stainless steel net, (GKD, Düren, Germany) planar Ni and Co foil, (Goodfellow, Bad Nauheim, Germany) planar Nichem 1151, (Atotech, Berlin, Germany) and Stellite 21 (Kennametal Stellite, Koblenz, Germany) were used as electrodes.

Gas chromatography was performed with a Shimadzu GC-2010 (Shimadzu, Japan) using a HP 5 column (Agilent Technologies, USA; length: 30 m, inner diameter: 0.25 mm, film: 0.25 mm, carrier gas: hydrogen; injector temperature: 250 °C; detector temperature: 300 °C (FID); column inlet pressure: 106 kPa). All GC measurements were performed starting with an initial of temperature of 50 °C. Heat rate was 10 °C/min ending up at 290 °C keeping this temperature for 15 min. Retention times of electrochemical degradation products and the applied internal standard (ISTD): vanillin (**1**) 10.5 min, acetovanillone (**2**) 11.64 min, dodecylbenzene (ISTD) 16.00 min. Internal standard calibration was performed following a one point calibration protocol [1].

Peristaltic pumping was performed using a Heidolph pump 5201 (Heidolph, Schwabach, Germany) equipped with a SP Quick pump head and a Viton® hose.

Errors were calculated according to the following equation with respect to the propagation of uncertainty. All parameters were expected to be independent.

$$\Delta m_p = \sqrt{\left(\frac{\Delta A_p \cdot m_{St} \cdot \varepsilon_i}{A_{St} \cdot m_i}\right)^2 + \left(\frac{A_p \cdot m_{St} \cdot \varepsilon_i \cdot \Delta A_{St}}{{A_{St}}^2 \cdot m_i}\right)^2 \left(\frac{A_p \cdot \Delta m_{St} \cdot \varepsilon_i}{A_{St} \cdot m_i}\right)^2 + \left(\frac{A_p \cdot m_{St} \cdot \Delta\varepsilon_i}{A_{St} \cdot m_i}\right)^2 + \left(\frac{A_p \cdot m_{St} \cdot \Delta\varepsilon_i \cdot \Delta m_i}{A_{St} \cdot m_i^2}\right)^2}$$

Δm_p = Error of formed product.

m_{St} = Mass of added ISTD.

Δm_{St} = Error of the mass of added ISTD (±3%).

m_i = Mass of initially applied substrate.

Δm_i = Error of the mass of initially applied substrate (±2 mg).

A_p = Integral of the product signal in the gas chromatogram.

ΔA_p = Error of the Integral of the product signal in the gas chromatogram (±2%).

A_{St} = Integral of the ISTD signal in the gas chromatogram.

ΔA_{St} = Error of the Integral of the ISTD signal in the gas chromatogram (±2%).

ε_i = Response factor for the corresponding analyte.

$\Delta\varepsilon_i$ = Error of the Response factor for the corresponding analyte (±2%).

Procedure for electrochemical degradation of lignin

A solution of 0.525 g Kraft lignin in 85 g 3 M NaOH was transferred into an undivided beaker type cell equipped with a heating jacket and the temperature was adjusted to 80 °C. Electrodes were dipped into the solution and a constant current electrolysis was performed. After application of the current the electrolysis was stopped and the mixture was allowed to cool to room temperature (approx. 22 °C). The pH of the reaction mixture was adjusted to 1–3 by addition of aqueous H_2SO_4 (50%). Precipitated lignin was removed by filtration over Celite™. Filter cake was rinsed

thoroughly with dichloromethane (approx. 40 mL) and the aqueous filtrate was extracted three times with dichloromethane (approx. 80 mL each portion). The combined organic fractions were washed with brine (approx. 50 mL) and dried with anhydrous sodium sulfate followed by solvent removal under reduced pressure. The organic residue was dissolved in 1 mL ethyl acetate, 2 µL of the ISTD dodecylbenzene were added and the sample was analyzed by gas chromatography.

A schematic set-up of an undivided beaker cell equipped with a heating jacket is depicted in Figure S1 [2].

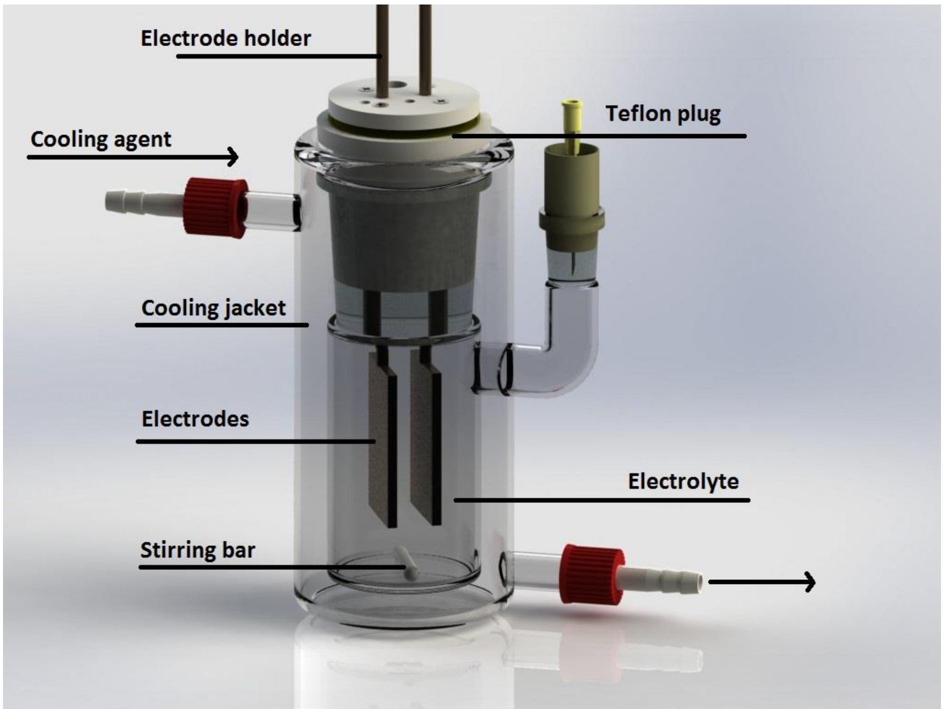

Figure S1: **Schematic set-up of an undivided electrolysis cell used for electrochemical degradation of lignin.**

Procedure for adsorption experiments with vanillin (1) model solutions (batch):

A solution of 50 mg vanillin in 85 g 3 M NaOH was transferred into a sealable vial and 1 g of the corresponding ion exchange resin was added. The suspension was placed on a plate vibrator and shaken for 60 min at 300 rpm. The loaded resin was removed by filtration from the depleted, alkaline solution (A) and thoroughly washed with distilled water (approx. 20 mL). Afterwards the resin was transferred into a sealable vial containing 50 mL of desorption solution (EtOAc/AcOH, ratio: 8:2). The suspension was shaken again for 60 min at 300 rpm. Depleted resin was removed by filtration and thoroughly rinsed with EtOAc (approx. 20 mL). Combined organic fractions represent the organic layer (B). Solution (A) was neutralized by addition of glacial acetic acid. The aqueous layer was extracted three times with EtOAc (approx. 50 mL each portion). The combined organic fractions were washed with 30 mL distilled water and dried with anhydrous sodium sulfate. Organic solvent was removed under reduced pressure. Organic residue was dissolved in 1 mL EtOAc, 2 µL of the ISTD dodecylbenzene were added and the sample was analyzed by gas chromatography. Organic layer (B) was washed with 30 mL distilled water. Solvent was removed under reduced pressure after drying with anhydrous sodium sulfate. The residue was dissolved in 6 mL EtOAc, 6 µL of the ISTD dodecylbenzene were added and the sample was analyzed by gas chromatography.

Procedure for adsorption in reaction mixtures after electrochemical degradation of lignin (batch)

The complete electrolyte after an electrochemical degradation of lignin according to the above mentioned procedure was transferred to a vial and Dowex Monosphere 550a OH was added to the solution. The suspension was placed on a plate vibrator and shaken for 60 min at 300 rpm. Loaded resin was removed by filtration from the depleted, electrolyte (A) and thoroughly washed with distilled water (approx. 20 mL). Afterwards the resin was transferred in a vial containing 50 mL of desorption solution (EtOAc/AcOH, ratio: 8/2). This suspension was again shaken for 60 min at 300 rpm. Depleted resin was removed by filtration and thoroughly rinsed with EtOAc (approx. 20 mL). Combined organic fractions represent the organic layer (B). Solution (A) was neutralized by addition of glacial acetic acid and precipitated lignin was removed by filtration using Cellite™. Filter cake was rinsed thoroughly with dichloromethane (approx. 25 mL). Aqueous filtrate was extracted three times with dichloromethane (approx. 80 mL each portion) and the combined organic fractions were washed with brine (approx. 50 mL). After drying of the organic layer over anhydrous sodium sulfate organic solvent was removed under reduced pressure. Brown residue was dissolved in 1 mL ethyl acetate and 2 µL of the ISTD dodecylbenzene were added and the sample was analyzed by gas chromatography. The organic fraction (B) were washed with 30 mL water. Solvent was removed under reduced pressure after drying over anhydrous sodium sulfate. Organic residue was dissolved in 1 mL EtOAc, 2 µL of the ISTD dodecylbenzene were added and the sample was analyzed by gas chromatography.

Procedure for continuous adsorption experiments

The corresponding amount of electrolyte after electrochemical degradation of lignin according to the above mentioned procedure was pumped through a column bed of Dowex Monosphere 550a OH. Bed volume (BV) of the resin was 17 mL and pumping speed 4 BV/h directed downwards. The column was washed after the adsorption process with 4 BV of distilled water at 4 BV/h directed downwards for neutralization. The loaded resin was treated with 5 BV of desorption solution (EtOAc/AcOH, ratio: 8/2) at a rate of 2 BV/h and reverse flow. The resulting organic layer was washed with approx. 50 mL distilled water and dried afterwards with anhydrous sodium sulfate. Organic solvent was removed under reduced pressure. Brown organic residue was dissolved in 8 mL ethyl acetate, 8 µL of the ISTD dodecylbenzene were added and the sample was analyzed by gas chromatography.

The experimental set up for continuous adsorption processes is shown in Figure S2.

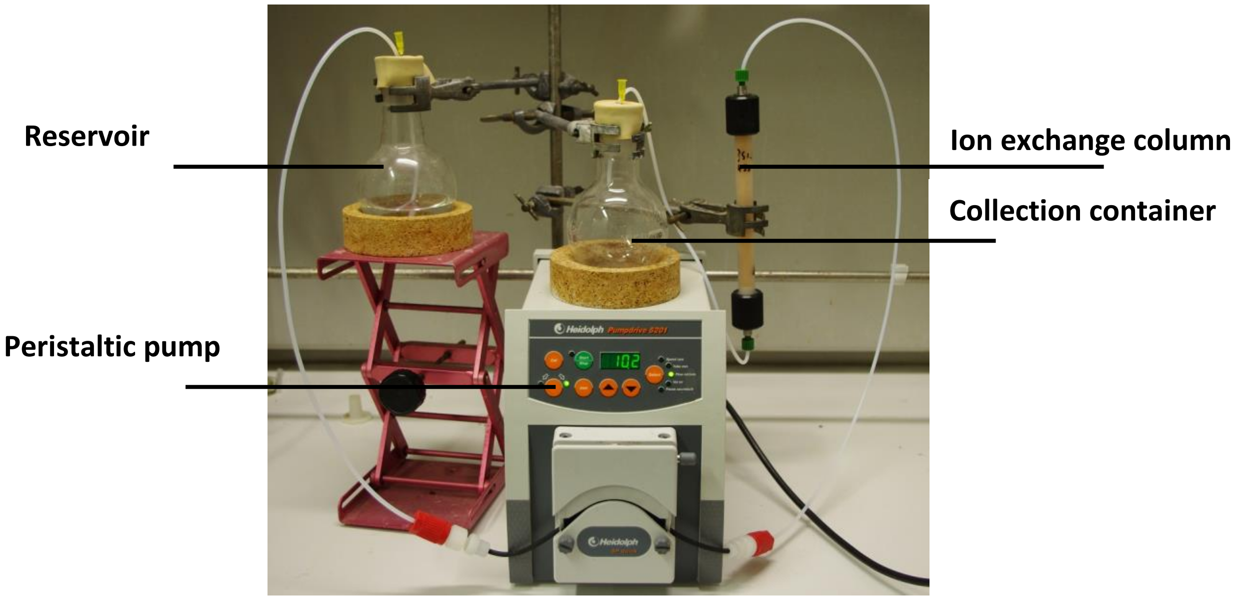

Figure S2: Experimental set up for continuous adsorption processes.

Procedure for electrochemical treatment of vanillin (1) in alkaline electrolyte

A solution of vanillin (**1**) in 85 g 3 M NaOH was transferred in an undivided beaker-type cell equipped with a heating jacket and the temperature was adjusted to 80 °C. Electrodes were dipped into the solution and a constant current electrolysis was performed at a current density of 38 mA·cm^{-2}. After application of the mentioned electricity the electrolysis was stopped and the mixture was allowed to cool to room temperature (approx. 22 °C). The pH of the reaction mixture was adjusted to 1-3 by addition of H_2SO_4 (half conc.). The aqueous solution was extracted three times with dichloromethane (approx. 80 mL each portion). The combined organic fractions were washed with brine (approx. 50 mL) and dried with anhydrous sodium sulfate followed by solvent removal under reduced pressure. The organic residue was dissolved in 1 mL ethyl acetate, 2 µL of the ISTD dodecylbenzene were added and the sample was analyzed by gas chromatography.

Results of the experiments are presented in Table S1. Gas chromatograms of the received product showing that vanillin (**1**) is the only observed component are depicted in Figure S3.

Table S1: Results of the electrochemical Treatment of vanillin (1) in alkaline solution and quantities of recovered starting material.

Entry	Va_i [mg]	Q [F]	Va_{re} [mg][c]
**1**[a]	5.43	58.0	4.95
**2**[b]	10.21	30.9	7.94

Conditions: 85 g 3 M NaOH, 80 °C, 38 mA·cm^{-2}; [a] = stainless steel net anode, [b] = Ni foam anode.

Va_i = Initially applied amount of vanillin (1), Va_{re} = Recovered amount of the starting material vanillin (1) after electrochemical treatment.

[c] = Quantification of the recovered starting material by ISTD method.

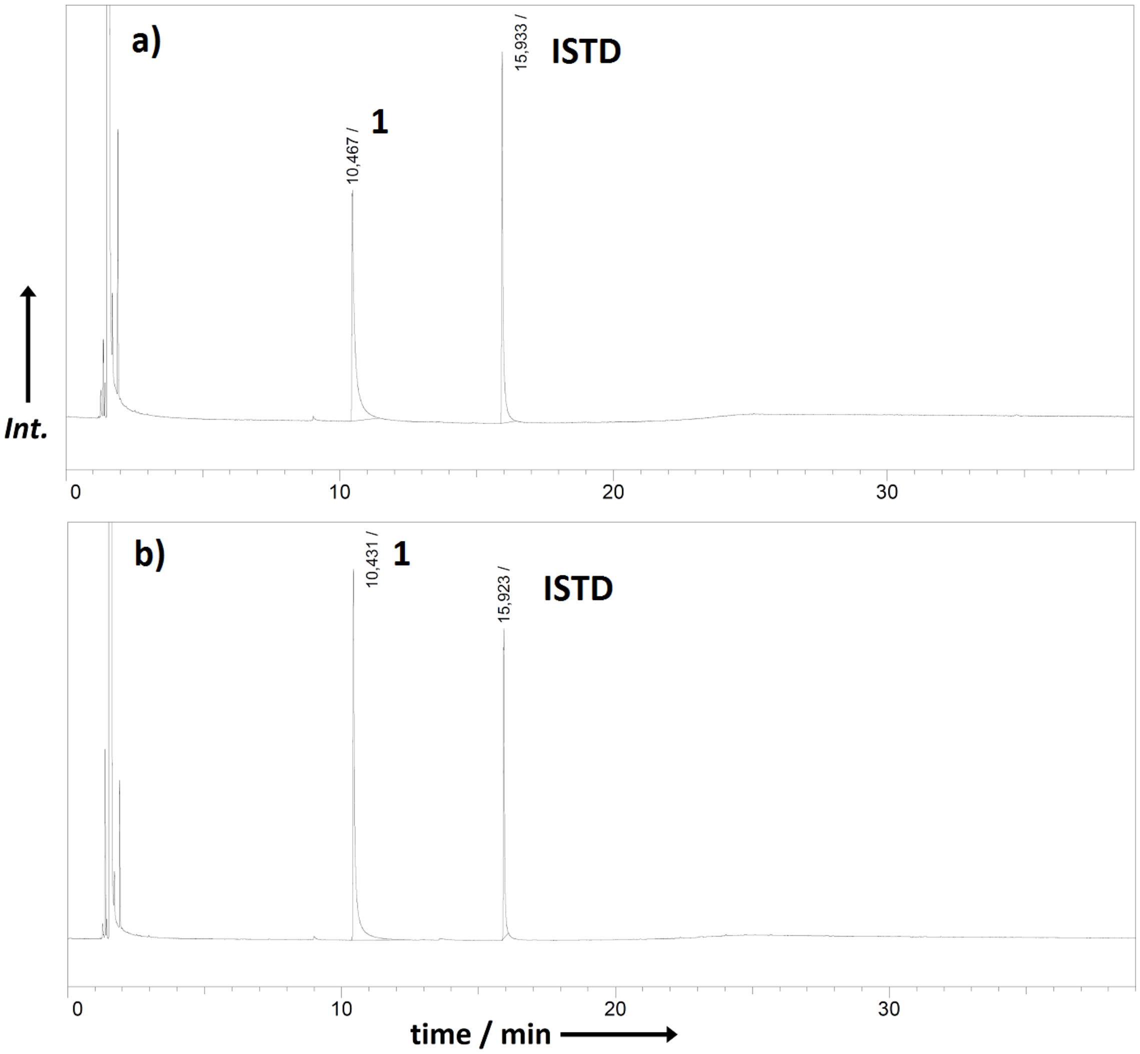

Figure S3: Gaschromatgrams of the products of electrochemical treatment of vanillin (1) in alkaline solution. a) Refers to entry 1 of Table S1. b) Refers to entry 2 of Table S1.

Quantification of the by-product acetovanillone (2) by ISTD method

Quantities for acetovanillone (**2**) are summarized in comparison to yields of vanillin (1) for representative experiments in Table S2. Experiments were performed according to the experimental procedure for electrochemical degradation of lignin in the manuscript.

Table S2: Yields of vanillin (1) and acetovanillone (2) by electrochemical degradation of vanillin at different anode materials.

Entry	Anode	1 [wt%][a]	2 [wt%][a]
**1**	Stellite 21	1.8	0.2
**2**	Ni (planar)	0.7	<0.1
**3**	Ni foam	1.1	0.1
**4**	Stainless steel net	0.9	0.1

Conditions: 0.525 g Lignin, 85 g 3 M NaOH, 80 °C, 1.9 mA·cm^{-2}, 2688 C·g^{-1}.

[a] = Yields were determined by ISTD method.

Quantification of vanillin (1) take up by adsorption on a gram scale and reactivation of the resin

A column was packed with 18.2 mL of Dowex Monosphere 550a OH (exchange capacity 1.1 meq). A 3.0 wt% solution of vanillin in 1 M NaOH was pumped through the bed at a rate of 4 BV·h^{-1} (BV·h^{-1} = bed volume per hour) until 1.1 equivalents, regarding the total exchange capacity of the resin, passed through the column (22.0 mmol, 3.34 g vanillin (**1**)). The adsorption is directed downwards. Afterwards

the column bed is washed neutral with H_2O (millipore grade, approx. 4 BV at a rate of 10 BV/h). For desorption the direction was changed to reverse flow. The system EtOAc:AcOH (8:2) was used for desorption of vanillin (**1**) and 7 BV of this solution were pumped through the bed at a rate of 2 $BV{\cdot}h^{-1}$. To avoid physisorbed residues of vanillin (**1**) on the resin the bed is treated further with 10 BV MeOH at a rate of 10 $BV{\cdot}h^{-1}$ and the organic fractions were combined and transferred to 500 mL volumetric flask (acidic fraction (A)). The resin was treated afterwards with 27 BV of 2.5 wt% aqueous NaCl, followed by 4.0 wt% NaOH at a rate of 7 $BV{\cdot}h^{-1}$ until no further Cl^{-}-ions were eluated (approx. 38 BV, test for chlorine ions using 0.1 M $AgNO_3$ solution). The column bed is washed neutral with H_2O (millipore grade, approx. 4 BV at a rate of 10 BV/h). The resin is then again treated with 27 BV of 2.5% aqueous NaCl and the aqueous eluent (B) is collected in a 500 mL volumetric flask. The volumetric flask containing the acidic fraction (A) was filled with MeOH and the solution was thoroughly homogenized. A 2 mL aliquot was taken and 2 µL of the ISTD dodecylbenzene were added. The sample was analyzed by gas chromatography and the total amount of desorbed vanillin (**1**) calculated by ISTD method. The volumetric flask containing the aqueous eluent (B) was filled with H_2O (millipore grade) and the solution was thoroughly homogenized. A 50 mL portion was taken and titrated with 0.1 M HCl against methyl red. The total exchange capacity of the resin was calculated by the consumed amount of 0.1 M HCl.

<u>Results of quantification and reactivation</u>

Results of the described experiment for the quantification of the maximum loading with vanillin (**1**) and the reactivation of the resin are summarized in Table S3.

Table S3: **Total amount of vanillin (1) desorbed from maximal loaded Dowex Monosphere 550a OH resin and exchange capacity after reactivation of the resin.**

Entry	x_i [meq]	Va_i [mmol]	Va_d [mmol]	$Va_{d,rel}$ [%]	x_d [meq]
**1**	20.2	22.0	13.28	65.7	20.3

x_i = Minimum exchange capacity of fresh resin (specification supplied by the producer), Va_i = Total amount of vanillin (1) passed through the column bed, Va_d = Total amount of vanillin (1) desorbed from the resin, $Va_{d,rel}$ = Loading of the resin with vanillin (1) related to the total exchange capacity, x_d = exchange capacity of the resin after desorption of vanillin (1).

References

1. Kuss, H. J.; Kromidas, S., (Eds.) *Quantification in LC and GC*. *A practical guide to good chromatographic data*, Wiley-VCH: Weinheim, Germany, **2009**. isbn:9783527323012

2. Schäfer, H. J. Electrochemistry in Radical Reactions. In *Radicals in Organic Synthesis*, Wiley-VCH: Weinheim, **2001**, pp 205-291.
